# Structural and Mechanical Properties of Recycled HDPE with Milled GFRP as a Filler

**DOI:** 10.3390/ma17235875

**Published:** 2024-11-29

**Authors:** Maciej Jan Spychała, Paulina Latko-Durałek, Danuta Miedzińska, Kamila Sałasińska, Iga Cetnar, Arkadiusz Popławski, Anna Boczkowska

**Affiliations:** 1 Faculty of Mechanical Engineering, Military University of Technology, Kaliskiego 2 St., 00-908 Warsaw, Poland; maciej.spychala@wat.edu.pl (M.J.S.); arkadiusz.poplawski@wat.edu.pl (A.P.); 2 Faculty of Materials Science and Engineering, Warsaw University of Technology, Wołoska 141 St., 02-507 Warsaw, Poland; paulina.latko@pw.edu.pl (P.L.-D.); kamila.salasinska@pw.edu.pl (K.S.); iga.cetnar.stud@pw.edu.pl (I.C.); anna.boczkowska@pw.edu.pl (A.B.)

**Keywords:** recycling, glass fibers, high-density polyethylene, mechanical properties, microstructure

## Abstract

The increasing complexity and production volume of glass-fiber-reinforced polymers (GFRP) present significant recycling challenges. This paper explores a potential use for mechanically recycled GFRP by blending it with high-density polyethylene (HDPE). This composite could be applied in products such as terrace boards, pipes, or fence posts, or as a substitute filler for wood flour and chalk. Recycled GFRP from post-consumer bus bumpers were ground and then combined with recycled HDPE in a twin-screw extruder at concentrations of 10, 20, 30, and 40 wt%. The study examined the mechanical and structural properties of the resulting composites, including the effects of aging and re-extrusion. The modulus of elasticity increased from 0.878 GPa for pure rHDPE to 1.806 GPa for composites with 40 wt% recycled GFRP, while the tensile strength ranged from 36.5 MPa to 28.7 MPa. Additionally, the porosity increased linearly from 2.65% to 7.44% for composites with 10 wt% and 40 wt% recycled GFRP, respectively. Aging and re-extrusion improved the mechanical properties, with the tensile strength of the 40 wt% GFRP composite reaching 34.1 MPa, attributed to a reduction in porosity by nearly half, reaching 3.43%.

## 1. Introduction

In a world of constantly growing demand for goods, the importance of plastic recy-cling became an essential aspect of the circular economy. Putting aside the fact that plastic recycling can limit the amount of litter polluting the environment, it also results in the possibility to reuse valuable materials from utilized objects. It is estimated that around 50% of produced thermoplastics are single-use ones, which are used for disposable plastic bags or food packaging [1]. A material that constitutes more than a third of the global market for thermoplastics is polyethylene (PE) [2], with over 103 million tons produced in 2016 [3]. It consisted of 46% high-density polyethylene (HDPE), which is used in the production of various types of bottles, durable plastic bags, and chemical and gasoline containers. It is also used as a 3D printing filament and as a material for flexible pipes and gaskets [4,5]. Nowadays, there is a lot of research on reinforcing or replacing raw material with recycled material, waste materials, or even organic fibers, like cellulose fiber. Such a composite can be more thermally stable than the HDPE matrix [6].

Currently, the predominant focus of public attention is directed towards the recycling of single-use plastics, with significant progress made in developing and implementing recycling procedures within industrial practices. This heightened emphasis on plastic recycling is attributed to the objectives outlined in the Green Deal policy established in Europe, which aims to achieve a 55% recycling rate for plastic packaging waste by 2030 [5]. While the recycling of simple plastic waste has been relatively straightforward, there exists a second category of polymeric waste that is generated in substantial quantities by society. This category encompasses composite materials, particularly fiber-reinforced polymers (FRP), which consist of long fibers such as glass or carbon embedded in a resin matrix, primarily polyester and epoxy. The non-biodegradable nature of synthetic fibers, coupled with the cured thermoset resin, presents significant challenges for recycling. In 2021, the European Composites market was valued at approximately 18 billion USD, with an anticipated growth rate of 5% during the period of 2022–2027. This growth is attributed to the escalating demand for lightweight materials, as well as mounting concerns regarding energy consumption across various industries including the wind, transportation, aerospace and defense, marine, and automotive sectors [7,8].

The most common approach of FRP recycling comprises mechanical and thermo-chemical processes. Former methods consisted of shredding, milling, or crushing composite structures, which were later washed and sorted to separate the coating and matrix from the fibers [9,10,11]. Newer methods focus on decomposing the matrix by the pyrolysis or fluidized bed method, amongst many others. Pyrolysis is a method that allows the thermal degradation of the matrix, while the fluidized bed method is based on using hot steam to decompose the matrix material [10,12]. Nevertheless, all the mentioned methods have their limitations. Mechanical decomposition evokes a high decrease in mechanical properties of fibers [9,13], in contrast to thermochemical methods. Lewandowski et al. [14] observed the increase in the elastic modulus and decrease in the tensile and impact strength of glass fibers with a carbon deposit, derived from the pyrolytical recycling. In addition, it must be noticed that the proposed and presently used recycling of glass fibers needs a complicated and energy-absorbing chemical or/and thermal processing. It can be hard to implement in industrial conditions and bring high costs [15]. Several noteworthy articles have been outlined in the extensive review performed by Scaffaro et al. [15] regarding the use of recycled glass and carbon fibers. Studies of great significance were also carried out by Evens et al. [16] and Kratz et al. [17], who, respectively, described the influence of mechanical recycling on the properties of fiber-reinforced polypropylene and evaluated the possibility of combining fibers from production waste with virgin fibers in the manufacturing of composites.

One potential method for reusing FRP involves extracting the fibers from the resin. Another option is to directly use the composite scraps after mechanical recycling, thereby eliminating additional energy consumption and utilizing the entire waste material. However, it is important to note that the presence of resin or paint traces may impact the final properties. Despite this, there is a discernible interest in this approach. Especially, milled GFRP waste originating from the pultrusion [18] and molding process [19] into the powder form has been used as reinforcement for concrete. It was pointed out that the very fine powder of GFRP waste can improve the concrete total workability expressed by the higher mechanical parameters and water absorption requirement of a fresh state and a reduction in pores in hard state concrete. The waste of FRP can be also used as a replacement for the calcium carbonate filler in sheet molding compounds, in artificial wood-based products, as reinforcement for the new FRP, as a filler for bitumen in road construction, or as a replacement of natural sand in mortar [19,20,21,22,23]. Much less attention is put on the implementation of recycled FRP particulates in the thermoplastic matrices. Butenegro et al. presented the composites of Polyamide 11 and Polyamide 12 with pultruded carbon -fiber reinforced polymer (CFRP) waste by the thermal pressing method [24].

The literature overview indicates a gap in the research on composites based on recycled thermoplastic polymers and FRP waste. To address this gap, the goal of the current study is to critically assess the feasibility of directly using GFRP waste after mechanical recycling in the recycled matrix. This assessment involves a comprehensive analysis of the mechanical properties of composites containing varying percentages of GFRP waste, coupled with microstructure observations. Additionally, the study investigates the post-processing and aging effects on the mechanical performance of the composites, aiming to provide insights into potential methods for integrating components from post-consumer sources.

## 2. Materials and Methods

### 2.1. Composites Manufacturing

**Figure 1:**
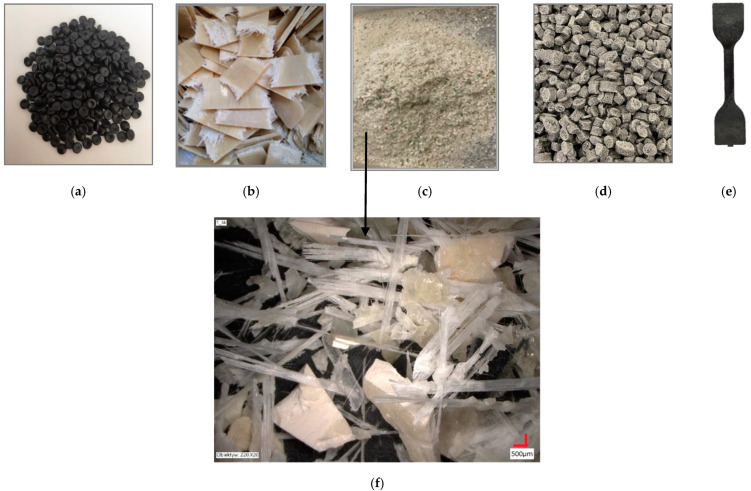
Tested material preparation process: (**a**) pellets of rHDPE; (**b**) bus bumpers waste; (**c**) rGFRP after grinding; (**d**) composite pellets 60% rHDPE + 40% rGFRP; (**e**) dog-bone shaped specimen for mechanical tests; and (**f**) rGFRP scraps at higher resolution.

The initial components were supplied from the market: commercial HDPE regranulate (rHDPE) (Figure 1a), having black color, with 0.9% of inclusions (average area S_z_ = 22 μm^2^; maximal area S_z_max_ = 450 μm^2^); and post-consumer glass-fiber reinforced polyester composite used as bus bumpers (Figure 1b), after mechanical recycling (rGFRP) from AGA Kompozyty Sp. z o.o. company (Pisarzowice, Poland). The mass fraction of fibers in the waste was determined by calculating the weight difference of the sample before and after burning off the resin. Share of glass fiber is 18 wt%. Because the delivered rGFRP waste has a diameter greater than 5 mm, therefore, to enable its trouble-free dosing into the extruder, they were reground by a mechanical shredder with 5 mm sieves (Figure 1c). A received mixture of mainly glass fibers (Figure 1f), cured resin and paint were used in unchanged form (without separation of each fraction) as the filler for rHDPE. Regarding complex issue of studying material properties of recycled composite materials with recycled multi-fracture filler, the authors followed phenomenological approach. Due to that fact, the material was treated as postproduction waste derived straight from the manufacturer and was not separated into different fractions. Moreover, rHDPE was chosen despite it having inclusions.

**Figure 2:**
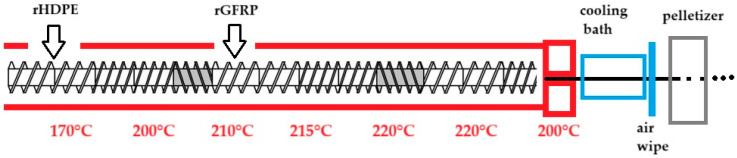
Scheme of material preparation.

Compounding was carried out by TMBK Partners Sp. z o.o. (Warsaw, Poland) using co-rotating twin-screw extruder, screw diameter 22 mm, L/D = 42, and 6 heating zones (temperature profile: 170 °C, 200 °C, 210 °C, 215 °C, 220 °C, 220 °C, and 200 °C for a die head). rGFRP scraps were introduced into the melted rHPDE by a side feeder at 3rd zone (Figure 2).

Subsequently, the melt was passed through a two-strand granulation head, then the strands were cooled with water and wiped by air curtain and, finally, cut on a knife pelletizer to the form of granules. During extrusion, an increase in melt pressure was observed with increasing concentration of the filler: from 0 bar up to about 80 bar. The mixing segments of screws, located in zones 4 and 5, caused the greater shear; hence, a temperature increase was observed. These zones were automatically cooled to maintain the set temperature and did not lead to overheating. Four composite types were produced in the form of the pellets (Figure 1d), with 10 wt%, 20 wt%, 30 wt%, and 40 wt% of rGFRP. Over 40 wt% material was unstable during the extruding process. For a concentration of 10 wt% rGFRP, the length of the granules is shorter than for the others; it results from a higher setting of the rotational speed of the granulator knife. The color of the pellets also changes from dark gray to light gray as the rGFRP content increases and visible white dots from polyester resin are visible (Figure 1d). Due to the porous structure of the granules, it absorbed a great amount of water, which is why it was pre-dried in vacuum of 35 mbar at 80 °C within 24 h and, finally, dried in compressed air dryer at 80 °C within 1 h. After drying, pellets were injected and molded into the dog-bone-shaped specimens [25] (Figure 1e) for the mechanical tests (length 74 mm, and thickness 3.89 mm), and cylinders for the tomography test (diameter 5 mm, and thickness 5 mm) using a HAAKE Mini Jet Piston Injection Molding System (ThermoFischer Scientific, Waltham, MA, USA). The parameters of the injection molding were as follows: 220 °C—the temperature of the barrel, 40 °C—the mold temperature, 700 bars and 7 seconds—injection pressure and time, and 600 bars and 5 seconds—the post-processing injection pressure and time.

### 2.2. Post-Processing and Aging

The composite of rHDPE with 40 wt% rGFRP was post-processed in a twin-screw extruder at 190 °C and screw velocity of 30 rpm using HAAKE MiniLab, ThermoFisher Scientific, Waltham, MA, USA. They also underwent the aging test at 60 °C, with humidity of 85% for 28 days at MKF240 climate chamber (Binder, Tuttlingen, Germany). The dog-bone-shaped specimens were weighted every 4 days to check their mass stability.

### 2.3. FTIR

Fourier-Transform Infrared Spectroscopy (FTIR) was applied to validate the composition of the rHDPE and rGFRP. For rHDPE, the analysis was made directly on the pellet; however, for rGFRP, only cross-linked resin and paint scraps were selected. Each sample was scanned 64 times with a resolution of 4 cm^−1^ in the frequency range 4000–400 cm^−1^.

### 2.4. Microstructure

The microstructure of rHDPE composites with various rGFRP concentration was analyzed directly on the pellets after extrusion using a Scanning Electron Microscope (SEM 3000 Hitachi, Tokyo, Japan). Pellets were coated with a thin layer of gold before measurement. The applied voltage was 5 kV for all materials. The porosity of the composites was analyzed using computed tomography (CT), and a SkyScan 1174 X-ray scanner (Bruker, Kontich, Belgium) on a cylindrical specimens from the injection molding process. The scanning parameters were as follows: image pixel size 20.90 μm, rotation step 0.100 deg, and frame averaging 3. Because of low density of the tested material, the 50 mm aluminum filter was used. For each composite and the reference sample (100% rHDPE), the specimens were scanned 3 times to calculate the average porosity using SkyScan software (NRecon 1.15 version and CTAnalyser version 1.15).

A spectrometer, an energy-dispersive spectrometer (EDS), that allowed for quantitative and qualitative analysis chemical composition of characteristic areas was used during SEM analysis with Hitachi Su-70 (Hitachi, Tokyo, Japan), 15 kV.

Optical microscopy studies using Keyence VHX-1000 (Keyence, Osaka, Japan) were conducted on bone-shaped samples prior to the tensile test. A cross-section was carried out at a distance of 1 cm from the edge of the handle section of the sample, followed by sanding with abrasive paper and polishing with a diamond suspension achieving a finish of 5 μm.

### 2.5. TGA

The thermogravimetric analysis (TGA) was employed to study the thermal stability of the composites in an inert atmosphere (nitrogen) and air. Samples of 10 ± 0.5 mg were placed in platinum crucibles and heated in a temperature range from ambient temperature to about 1000 °C, with a 10 °C/min rate, using a TGA Q500 (TA Instruments, New Castle, DE, USA) apparatus.

### 2.6. DSC

The differential scanning calorimetry (DSC) was carried out using a DSC Q1000 device (TA Instruments, New Castle, DE, USA). Samples of 5 ± 0.1 mg were placed in sealed aluminum crucibles, which were cooled down to −80 °C and heated to 200 °C at a rate of 10 °C/min. The analysis was performed in the heating/cooling/heating cycles, and the results were analyzed using the Universal Analysis 2000 ver.4.5A software (TA Instruments, New Castle, DE, USA). The crystallinity degree *X_c_* of the samples was calculated using Formula (1):

(1) $$X_{C}=\frac{\Delta H_{m}}{\left(1-\varphi \right)\cdot \Delta H_{m}^{o}}\cdot 100\%$$

where Δ*H_m_*—melting enthalpy of a sample, Δ*H_m_*_100_—melting enthalpy of 100% crystalline polyethylene, $\Delta H_{m}^{o}$ = 288 J/g [26], and *φ*—filler weight fraction.

### 2.7. Mechanical Properties

The purpose of the test was to determine the mechanical characteristics of the rHDPE/rGFRP composites before and after aging. The test samples were made in the injection mold, which allowed for high accuracy of geometric fittings (Figure 1e). Thickness and width of samples in the measuring part were 2.02 mm and 3.89 mm, respectively. The quasi-static tensile test was carried out with the use of the electromechanical Zwick/Roell Kappa 50 DS testing machine (ZwickRoell, Ulm, Germany) equipped with the 50 kN measurement head. The applied uniform load speed was 5 mm/min in each test according to standard ASTM [27]. Data recording was carried out with the 20 Hz sampling frequency. The tests were carried out in room temperature.

## 3. Results and Discussion

### 3.1. FTIR Analysis

**Figure 3:**
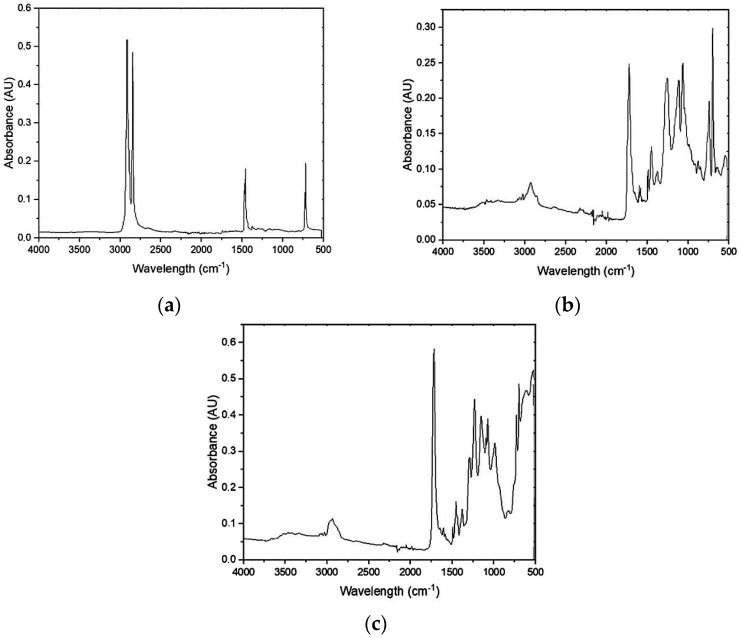
FTIR spectrum of (**a**) rHDPE; (**b**) resin from rGFRP waste; and (**c**) white paint from rGFRP waste.

To confirm that rHDPE does not contain any other plastics, the FTIR analysis was performed. The FTIR spectrum showed in Figure 3a coincides with the HDPE because of the characteristic peaks related to the chemical structure of the HDPE polymer. The peaks at the wavelengths 2913 cm^−1^ and 2846 cm^−1^ are related to the -CH_2_- asymmetric stretch and -CH_2_- symmetric stretch, respectively. The split peak in the region of 1462 cm^−1^–1471 cm^−1^ corresponds to the balance-type deformations of the -CH_2_- in the main polymer chain, whereas the split peak at 718 cm^−1^–729 cm^−1^ comes from the bond rocking of the methylene groups in phase and out of phase. The FTIR analysis of bus bumper waste (rGFRP) was made for the cured resin and paint residue. For the resin (Figure 3b), there are visible peaks at 1493 cm^−1^ and at 697 cm^−1^ which correspond to the presence of aromatic carbons. The peaks at 1462 cm^−1^ and 2000 cm^−1^ both indicate the presence of aromatic rings. Apart from that, there is also a peak at 1720 cm^−1^ which indicates a double bond C=O.

The analysis confirms that the resin was a polyester polymerized from the styrene monomer. Moreover, the observable peaks can also correspond to the presence of butyl benzyl phthalate, commonly used as a plasticizer for polyester resin. An FTIR spectrum of paint (Figure 3c) has peaks coming from the vibrations of C=O and C-H in the aromatic rings at 1720 cm^−1^, 1493 cm^−1^, and 697 cm^−1^. There is also a strong peak at 1275–1200 cm^−1^ suggesting C-O bond stretching typical for alkyls and a peak in between 2840–3000 cm^−1^ corresponding to the stretching vibration of alkyl C-H. According to the analysis, most probably, the paint contains poly(diallyl isophthalate), which is used in the coating and painting industry and has water resistance properties. It should be emphasized that it was not possible to fully separate the rest of the paint and resin; therefore, the used rGFRP contains a small amount of them.

### 3.2. Microstructure Study Results

**Figure 4:**
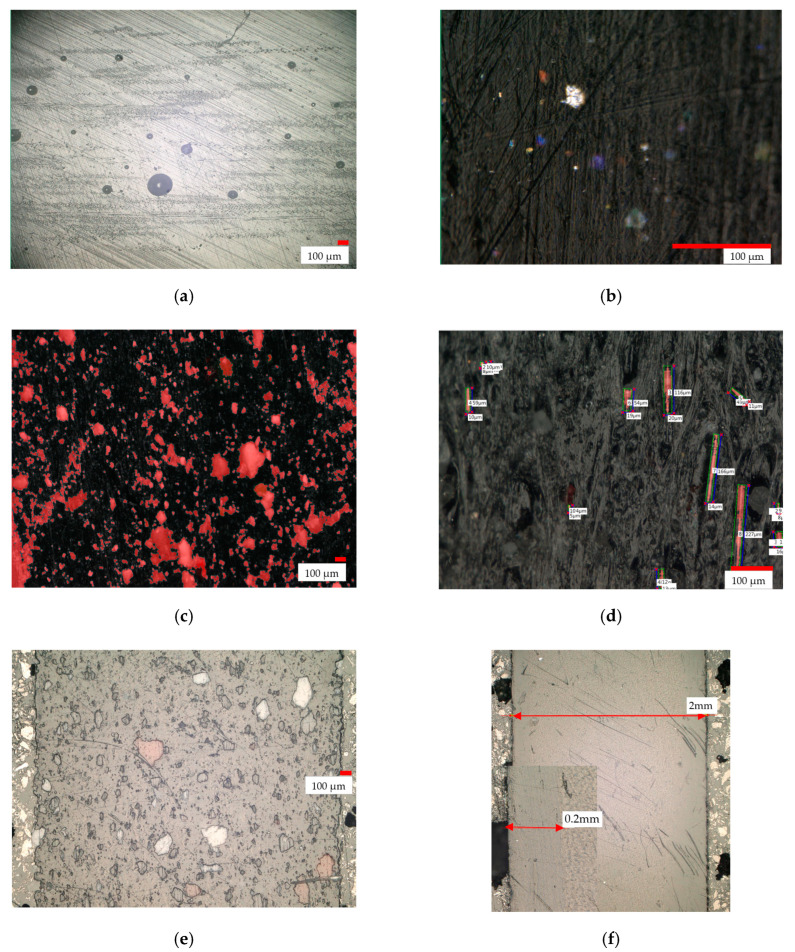
Microstructure observations: (**a**) image of cross-section of rGFRC material before milling; (**b**) image taken by Keyence VHX-1000 microscope of surface of the rHDPE pellets; (**c**) example of image of filler contamination analysis of rHDPE/rGFRC 40% sample; (**d**) example of image with fiber glass contamination analysis of rHDPE/rGFRC 40% sample; (**e**) surface of cross-section of rHDPE/rGFRC 40% sample; and (**f**) outer layer of rHDPE material with dimension.

The macro view of rHDPE/rGFRP shows large fragments of resin in the structure (Figure 4e). Based on the studies of 35 cross-section pictures (Figure 4c,d), some parameters were determined: *L_Aw_* = 0.0011 μm^−1^ (the total length of fibers divided by the total area), *S_Aw_* = 1.1% (the percentage area share of fibers); *L_Aw_* = 18.3% (the average percentage area share of the resin); *L_Aw_max_* = 33 μm^2^ (the average area of the resin fragment); and *L_Aw_max_* = 506 μm^2^ (the largest area of the resin fragment).

During the molding process, the outer layer of the rHDPE material was formed. The thickness of the layer is 200 µm (Figure 4f). In the sample rHDPE/rGFRP, such a layer was not formed (Figure 4e). The surface of the composite sample is rough

Based on the pictures, the distribution of the filler is random; agglomerates are observed.

SEM images of the composites made for various ratios of rHDPE and rGFRP at a magnification ×100 and ×500 are collected in Table 1. At the magnification of ×100, the general structure of the material can be seen. The smaller the percentage of glass fiber in the sample, the more compact the structure and more regular the placement of glass fibers. It was also clearly visible that the pores in the material structure were present in all of the samples. Moreover, the structural integrity of samples containing a higher percentage of glass fibers was compared. At the magnification of ×500, it is possible to see other fragments of rGFRP prevalent in the sample, such as paint or resin. Their presence can affect the integrity of the entire structure, and, therefore, the physical properties of composites. Most of the particles derived from rGFRP are placed near large pores in the structure. It is also possible to see the length and the orientation of single glass fibers. The higher the ratio between rHDPE and glass fibers, the longer the glass fibers that are present. In addition, glass fibers are randomly dispersed in all composites, which causes the isotropic properties of the material. However, the connection between HDPE and glass fibers in the samples with fewer glass fibers is more durable as the particles are firmly combined.

**Figure 5:**
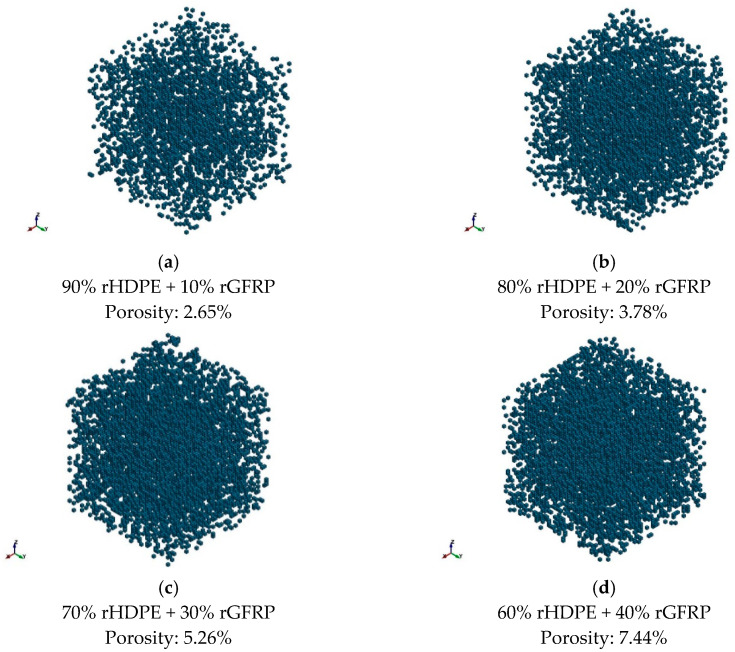
Results of CT for composites of rHDPE containing (**a**) 10 wt%; (**b**) 20 wt%; (**c**) 30 wt%; and (**d**) 40 wt % of rGFRP.

**Figure 6:**
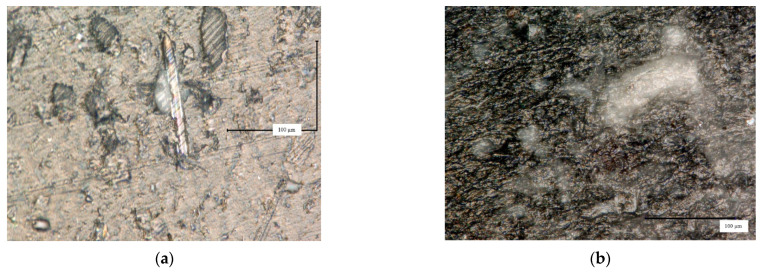
Images of rHDPE/rGFRP material: (**a**) cross-section; and (**b**) surface of the sample.

To assess the porosity of the achieved composites, the results of CT scanning were presented in Figure 5. The linear increase in porosity coupled with the glass fiber content was observed. Similar characteristics for glass fiber composites were achieved by Monticeli et al. [28] and Lekube et al. [29]. The pores appeared especially in the surroundings of glass fibers. The same effect is visible in the SEM results in Table 1. However, the distribution of pores is almost uniform because of the uniform distribution of glass fibers (Figure 6). The porosity can be caused by the lack of adhesion between rHDPE and rGFRP as well as by the inclusions in the form of polyester resin. The intermediate layers between polyester resin and rHDPE are not perfectly connected [30].

A better interface can be observed when looking on the surface of the aged samples; the reason is the degradation of the polymer chains and reduction in pores (Figure 6).

**Figure 7:**
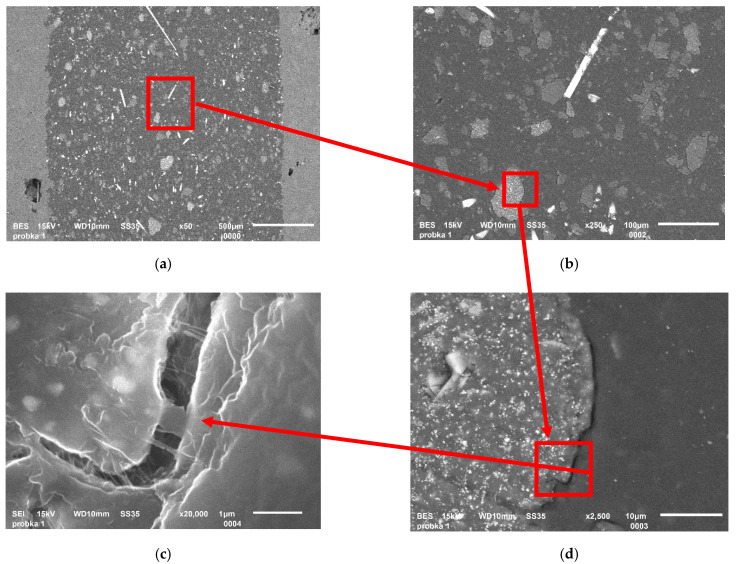
Images of rHDPE/rGFRC material: (**a**) macro view on the image; and (**b**–**d**) close-up of selected area—brighter areas are elements with higher mass number.

It was found that the resin contains fragments that are metallic elements such as titanium, magnesium, and aluminum (Figure 7). The occurrence of the last one is a consequence of the soil contamination of rGFRP. The others were ground off the mill blades; hence, it occurs only in the resin.

### 3.3. Thermal Analysis

The thermal stability of composites was investigated via thermogravimetric analysis. The values of the 5% mass loss (T_5%_), the maximum thermal degradation intensity, and the rate of decomposition, as well as the residual mass in an inert atmosphere and air were specified. The results of the thermal degradation are presented in Table 2, as well as the TG and DTG curves. The waste rGFRP, as a composition of unsaturated polyester resins, short glass fibers (10–25% by volume), colorants, fire retardants, and fillers, including calcium carbonate, presented numerous stages of degradation [31,32].

The first peak at 202 °C resulted from water loss through dehydration, and the release of volatile products, as well as the decomposition of unsaturated polyester resin, which consists of the scission of the cross-link structure and the formation of styrene and linear polyester [33,34]. The double DTG peak observed at 340 °C and 375 °C corresponds with the degradation of the unsaturated polyester, resulting from the chain scission of polystyrene, as well as polyester fragments. Moreover, the degradation of the transient char at a temperature above 600 °C, a characteristic used mostly for the analysis carried out in oxygen conditions [35], can be observed for the initial rGFRP. The DTG of rHDPE showed only one peak at 480 °C, corresponding to the degradation of the polymer. Consequently, the thermal decomposition of composites proceeded in two steps observed at 385 °C and 474 °C (also high intensity at 476, for HDPE filled with cellulose fibers [7]), resulting from the degradation of the filler and matrix, respectively.

Both waste components exhibited a 70% mass loss at 800 °C for the analysis performed in nitrogen; however, rHDPE in the oxidizing atmosphere showed only 1% of residue. It confirms a significant share of inorganic substances in post-consumer waste, which impacted the yield of residues in composites, increased with the filler content. Moreover, a lower share of organic parts in a filler decreased the decomposition rate in separate stages (Table 2). For the unmodified polyethylene, a 5% weight loss temperature (T_5%_)_,_ corresponding to the decomposition onset temperature, reached 480 °C. The addition of rGFRP lowered the T_5%,_ and the values decreased as its amount increased. This is due to polyester resin’s lower thermal stability compared to polyethylene.

**Figure 8:**
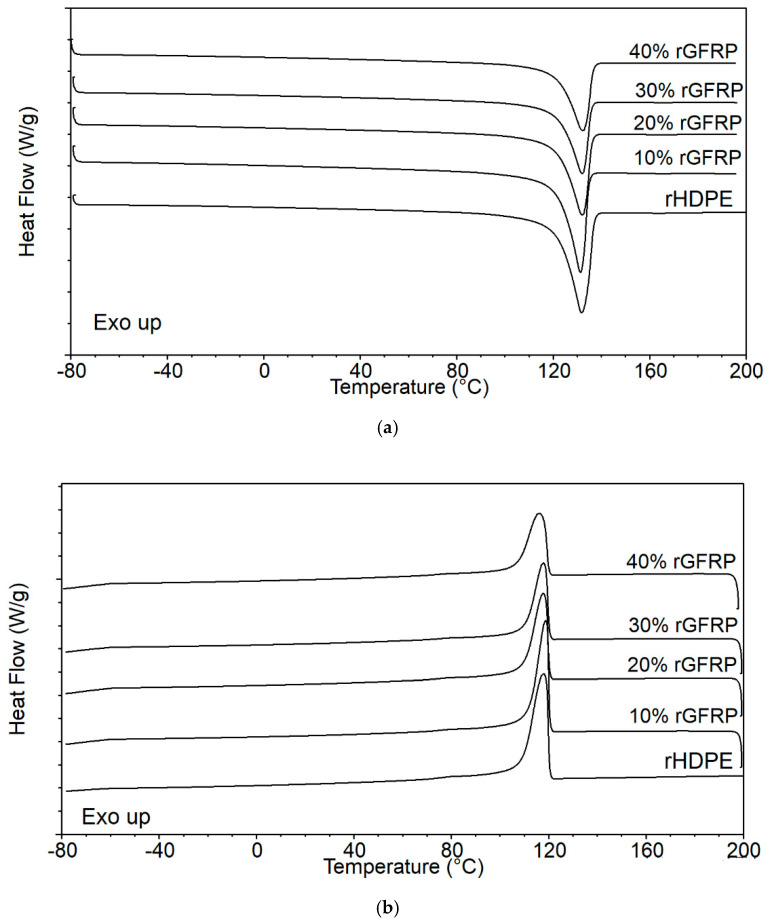
Behavior of rHDPE/rGFRP composites during (**a**) second heating, and (**b**) cooling, obtained by DSC analysis.

The impact of the rGFRP addition on the thermal properties of rHDPE composites was specified using calorimetric investigations. The changes in the crystallization (T_c_) and melting temperature (T_m_), as well as the enthalpy of fusion (ΔH_m_) as a function of the filler amount, are shown in Table 3. In turn, Figure 8 illustrates DSC thermograms received from DSC investigations during the first cooling and second heating.

rGFRP did not cause notable changes in the T_m_, whereas a slight downward was reported in the case of Tc for the sample with the highest amount of filler. The DSC cooling curve for the composites showed changes in the signal intensity compared to rHDPE; however, only for the composite with 40 wt% rGFRP were slight differences in the crystallinity content observed. Due to a relatively high number of side branches, rHDPE can form a crystalline structure, and the use of fillers does not usually lead to significant heterogeneous nucleation effects. Consequently, a lack of considerable changes in the composite’s crystallinity compared to the unmodified rHDPE was observed. Moreover, for all materials, a second barely noticeable peak at 75 °C, implying the presence of crystallites with various thicknesses, was noted. Usually, the double crystallization peaks reveal the effect of chain branches, which form less stable crystals at lower temperatures [26,36,37].

### 3.4. Mechanical Properties Study Results

**Figure 9:**
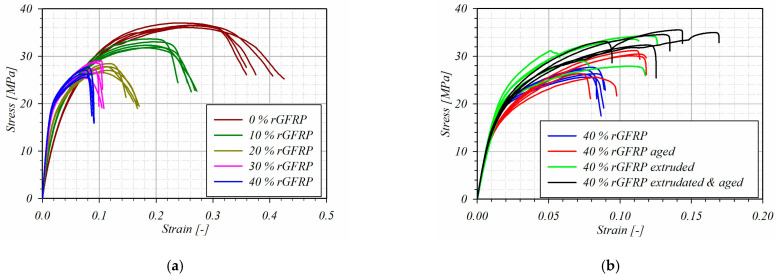
Engineering stress–strain curves for tensile test samples: (**a**) with varying percentages of rGFRP; and (**b**) with 40 wt% rGFRP after additional processes.

**Figure 10:**
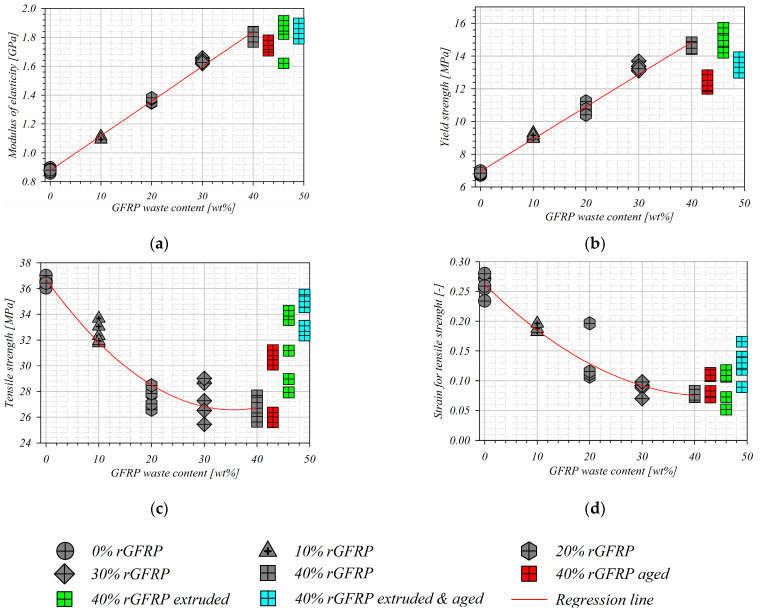
Comparison of (**a**) modulus of elasticity; (**b**) yield strength; (**c**) tensile strength; and (**d**) strain for tensile strength analyzed for all studied composites.

The direct results were achieved as the force–displacement relationship and were converted to engineering stress–strain curves considering the gage length of 25 mm. The modulus of elasticity E was calculated for the quasi-linear area of the stress–strain relationship (for a strain range of 0.0005 to 0.002) as a tangent of the slope of the straight line passing through the extreme point of the range. The offset yield point Re_0.2_ was calculated as a stress value which corresponds to a point at the intersection of a stress–strain curve and a line which is parallel to a specified modulus of the elasticity line shifted to a strain of 0.002. The tensile strength R_m_ was considered as a maximum stress value during the test, and, for this value, the strain for the tensile strength ε_Rm_ was described [27]. The results of the aforementioned parameters were presented and compered in Figure 9 and Figure 10. For all achieved results series, the average value and standard deviation were calculated and the results characterized by a gross error were rejected (Table 3).

The curves in Figure 10 mark the average values’ flow. First of all, it should be mentioned that the results’ deviation is acceptable for the mechanical testing of inhomogeneous materials [38]. The first verification of the achieved results was carried out for the recycled pure HDPE parameters. They were compared with the literature data. Murat et al. [39] studied the mechanical properties of HDPE acquired from the detergent bottle recycling process. This achieved the tensile strength of 25 MPa. The same result was acquired by Amjadi and Fatemi [40], but the authors concluded that the tensile behavior is strongly influenced by the processing and the temperature of testing. Values similar to those achieved in the presented study were shown by Blaise et al. [41]. Analyzing the elastic modulus change, it is visible that the increase is linear with the fiber content increase. The composite is characterized by a higher stiffness than the pure rHDPE. A similar linear nature of the course was observed for offset yield point values. Moreover, Farahana et al. [42] studied the mechanical properties of the rHDPE filled with ethylene vinyl acetate/eggshell powder. They achieved the similar parabolically decreasing characteristic of strength and elastic modulus vs. the share of filler. On the other hand, Malyuta et al. [43] studied the mechanical properties of the rHDPE/talc composite with 10–38 wt%, and concluded that the tensile strength and stiffness linear increased with a higher talc content. The elastic modulus increased by approximately 100% when the filler content reached 38% [43]. The composite is characterized by a higher stiffness than the pure rHDPE (the same tendency was presented by Ghernaout et al. for HDPE filled with cellulose fibers [7]). The stiffness improvement in the elastic region is caused by the high stiffness of glass fibers. The decrease in the tensile strength and the strain for it was observed and the character of this change was exponential. This is a very interesting phenomenon, because it differs from the literature results. AlMadeed et al. [44], Koffi et al. [45], Bajracharya et al. [46,47], and Singleton et al. [48] studied the reinforced recycled HDPE. They found the same relationship (the increase with a reinforcement increase) for the modulus of elasticity and offset yield point. The significant difference appeared when analyzing the tensile strength. In all papers mentioned above, the tensile strength increases with the reinforcement increase. The observed phenomenon can be caused by the fact that the used reinforcement was not specially prepared; the admixture of resin and acrylic was included in the prepared composite structure. In addition, no compounds were added. The decrease in tensile strength can be caused by the phase boundaries between the rHDPE, resin, and acrylic, and the low adhesion of the recycled fibers to the polymer matrix. A similar phenomenon was observed by Arunit et al. [49]. They proposed the additional post-curing in high temperatures to increase the tensile strength of the composites with the reinforcement made of not-processed milled glass fiber composites.

**Figure 11:**
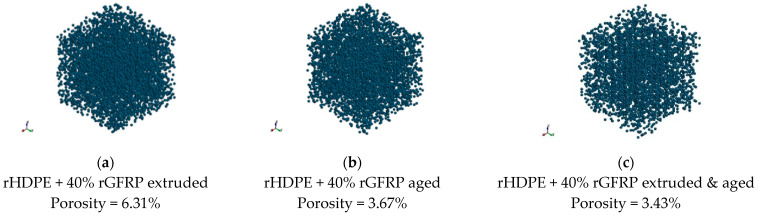
Results of CT for composites of rHDPE containing 40 wt% rGFRP after (**a**) additional extrusion; (**b**) aging; (**c**) and extrusion and aging. Measured region: 1 mm × 1 mm × 1 mm region, with resolution 20.90 m.

Next, the samples after aging, extrusion, and aging and extrusion processes with 40 wt% of rGFRP were mechanically tested. The results were also shown in Figure 9 and Figure 10, and in Table 3. It can be noticed that the additional processing did not influence the elastic modulus and yield strength. However, the improvement is visible for tensile strength, which is higher than for the unprocessed material and appears for a lower value of strain, similar to the results presented by Ayadi et al. [50]. This phenomenon appears especially for samples with 40% rGFRP extruded and aged. First of all, the reason for such phenomena is a porosity decrease, which was measured using CT tomography and presented in Figure 11. The porosity influences the tensile and compression strength, because, in the elasticity region, only the basic material surrounding the pores works [51]. The loss of porosity can be cause by the effect of repolymerization at the interface between rHDPE and polyester resin [52,53]. On the other hand, the simple post-curing process can lead to porosity reduction results in the case with a higher strength [54].

However, it must be noticed that the effect is opposite to the behavior of composites made only with glass fibers and plastics, for which the matrix degrades over time, leading to the formation of micro cracks [55].

## 4. Conclusions

In the presented research, the preparation process and structural and mechanical properties in the study of the composite made fully of recycled materials were presented. The composite was made of rHDPEand a milled polyester–glass composite with no additional thermal or separation treatment. The main goal was to assess the influence of various percentages of GFRP on the global mechanical properties of the final material. In addition, the influence of post-processing and aging was tested.

The FTIR analysis of chemical components showed that the used rHDPE did not contain any other plastics, while rGFRP was composed of a matrix made of polyester polymerized from the styrene monomer, glass fibers, and paint made of (diallyl isophthalate).

The microstructural analyses carried out using SEM and CT scanning revealed a significant level of porosity, especially in the surroundings of glass fibers, which could be caused by the lack of adhesion between rHDPE and rGFRP, as well as by the inclusions in the form of polyester resin. In addition, it was observed that glass fibers are less unidirectionally placed in the samples with a higher amount of glass fibers, which is linked to the isotropic properties of the material. However, the connection between HDPE and glass fibers in the samples with fewer glass fibers is more durable as the particles are firmly combined.

Moreover, the thermal properties of the achieved composites were studied. In this structural analysis, a lack of considerable changes in the composite’s crystallinity compared to the unmodified rHDPE, as well as the presence of crystallites with various thicknesses were observed.

To study the mechanical properties of composites, the quasi-static tensile test was carried out. We noticed a higher stiffness and yield point values of composites compared to the pure rHDPE. Next, the exponential decrease in the tensile strength and the strain with the increase in rGFRP content was observed, which could be caused by the fact that the used reinforcement was not specially prepared; the admixture of resin and acrylic was included into composite structure. Observing the SEM images, it can be concluded that the decrease in tensile strength can be caused by the phase boundaries between the rHDPE, resin, and acrylic, and the low adhesion of the recycled fibers to the polymer matrix.

To study the influence of processing and environmental conditions, the composite with 40% of rGFRP was directed towards the aging, extrusion, and aging and extrusion processes. A significant change in mechanical properties was observed. The improvement was noticed for tensile strength, which is higher than for unprocessed material and appears for lower values of strain. The phenomenon can be explained with the tomography results, which showed that the porosity of the aged composite decreased, which could be caused by the effect of repolymerization at the interface between rHDPE and polyester resin.

Moreover, we can significantly reduce the material costs for potential applications. Manufacturers producing glass-fiber-reinforced composites typically pay around €300 per 1000 kg to dispose of post-production waste, while HDPE costs about €909 per 1000 kg. By replacing 40 wt% of HDPE with rGFRP, material costs can be reduced by approximately 27%. Finally, it can be concluded that the aim of the paper was reached. The composite made of rHDPE and rGFRP with no additional treatment like the thermal removal of resin from GFRP was prepared. The amount of rGFRP content of 10 wt%, 20 wt%, 30 wt%, and 40 wt% was reached. The mechanical properties of the composites were studied and it was shown that they strongly depend on the microstructure of the material. The modulus of elasticity varied from 0.878 GPa for pure rHDPE to 1.806 GPa for the composite with a 40 wt% rGFRP content, when the tensile strength changed from 36.5 to 28.7 MPa. The porosity of the achieved composites increased from 2.65 (rGFRP content of 10 wt%) to 7.44% (rGFRP content of 40 wt%). In addition, it was shown that the extrusion and aging positively affected the mechanical properties (e.g., the tensile strength for a composite with an rGFRP content of 40 wt% reached 34.1 GPa) when the porosity decreased because of repolymerization at the interface between rHDPE and the polyester resin (3.43%).

## Figures and Tables

**Table 1:** SEM images of rHDPE/rGFRP composites.

Composite	Magnification ×100	Magnification ×500
90% rHDPE + 10% rGFRP	* 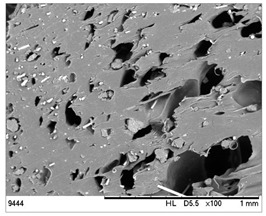 *	* 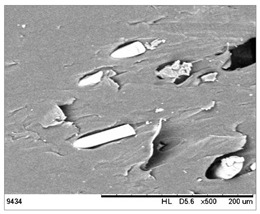 *
80% rHDPE + 20% rGFRP	* 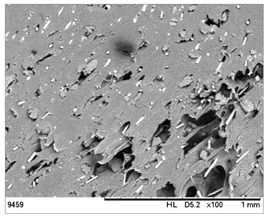 *	* 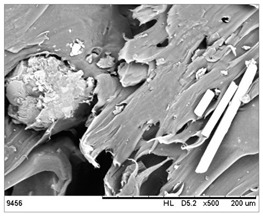 *
70% rHDPE + 30% rGFRP	* 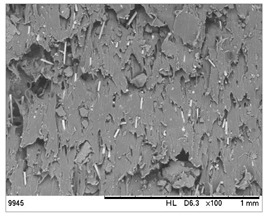 *	* 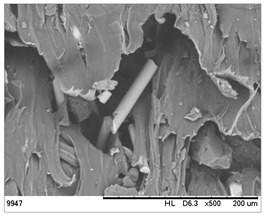 *
60% rHDPE + 40% rGFRP	* 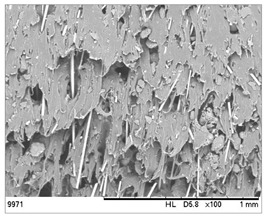 *	* 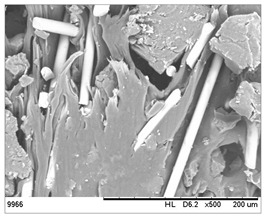 *

**Table 2:** TG and DTG data of rHDPE, rGFRP, and rHDPE/rGFRP composites.

Material	T_5%_	1st DTG Peak	2nd DTG Peak	3nd DTG Peak		4nd DTG Peak	Residual Mass at 800 °C (Nitrogen)	Residual Mass at 800 °C (Air)
	°C	°C; %/°C					%	
rGFRP	221	202; 0.1	340; 0.5	375; 0.6	-	611; 0.01	29.5	31.8
rHDPE	462	-	-	-	481; 3.5	-	30.8	1.4
rHDPE + 10% rGFRP	349	-	-	383; 0.1	475; 2.6	-	5.9	1.2
rHDPE + 20% rGFRP	333	-	-	386; 0.2	475; 2.0	-	12.3	9.0
rHDPE + 30% rGFRP	326	-	-	385; 0.2	473; 1.9	-	9.8	10.0
rHDPE + 40% rGFRP	327	-	-	386; 0.2	474; 1.8	-	15.8	12.5

**Table 3:** Results of statistical analysis of parameters calculation—average values with standard deviation.

Composite	Modulus of Elasticity E [GPa]	Offset Yield Point Re_0.2_ [MPa]	Tensile Strength R_m_ [MPa]	Strain for Tensile Strength ε_Rm_ [-]
0% rGFRP (rHDPE)	0.878 ± 0.014	6.8 ± 0.1	36.5 ± 0.3	0.260 ± 0.016
10% rGFRP	1.101 ± 0.010	9.1 ± 0.1	32.6 ± 0.7	0.189 ± 0.006
20% rGFRP	1.363 ± 0.014	10.7 ± 0.3	27.5 ± 0.7	0.129 ± 0.034
30% rGFRP	1.639 ± 0.016	13.3 ± 0.2	27.4 ± 1.3	0.088 ± 0.010
40% rGFRP	1.806 ± 0.026	14.6 ± 0.1	26.6 ± 0.7	0.077 ± 0.004
40% rGFRP aged	1.746 ± 0.025	12.3 ± 0.3	28.7 ± 2.3	0.097 ± 0.017
40% rGFRP extruded	1.813 ± 0.102	15.0 ± 0.5	31.2 ± 2.5	0.092 ± 0.025
40% rGFRP extruded and aged	1.844 ± 0.036	13.4 ± 0.4	34.1 ± 1.2	0.129 ± 0.017

## Data Availability

The data presented in this study are available on request from the corresponding author due to Military University of Technology Data Safety Policy.

## References

[1] Kehinde O., Ramonu O.J., Babaremu K.O., Justin L.D. (2020). Plastic wastes: Environmental hazard and instrument for wealth creation in Nigeria. Heliyon.

[2] Ronca S. (2017). Chapter 10—Polyethylene. Brydson’s Plastics Materials.

[3] (2024). Polyethylene (PE) Production Distribution Worldwide in 2016, by Region. https://www.statista.com/statistics/858191/global-polyethylene-production-distribution-by-region/.

[4] Tiago G.A.O., Mariquito A., Martins-Dias S., Marques A.C. (2023). The problem of polyethylene Waste—Recent attempts for its mitigation. Sci. Total Environ..

[5] Kazemi M., Kabir S.F., Fini E.H. (2021). State of the art in recycling waste thermoplastics and thermosets and their applications in construction. Resour. Conserv. Recycl..

[6] Ghernaout D., Belaadi A., Boumaaza M., Chai B.X., Jawaid M., Abdullah M.M.S., Krishnasamy P., Al-Khawlani A. (2024). Effects of incorporating cellulose fibers from *Yucca treculeana* L. on the thermal characteristics of green composites based on high-density polyethylene: An eco-friendly material for cleaner production. J. Mater. Res. Technol..

[7] Plastic Waste and Recycling in the EU: Facts and Figures|News|European Parliament 2018. https://www.europarl.europa.eu/news/en/headlines/society/20181212STO21610/plastic-waste-and-recycling-in-the-eu-facts-and-figures.

[8] Job S. (2013). Recycling glass fibre reinforced Composites—History and progress. Reinf. Plast..

[9] Dorigato A. (2021). Recycling of thermosetting composites for wind blade application. Adv. Ind. Eng. Polym. Res..

[10] Utekar S., More N., Rao A. (2021). Comprehensive study of recycling of thermosetting polymer Composites—Driving force, challenges, and methods. Compos. Part B Eng..

[11] Pimenta S., Pinho S.T., Worrell E., Reuter M.A. (2014). Recycling of Carbon Fibers. Handbook of Recycling.

[12] Zhang Y., Pontikes Y., Lessard L., Van Vuure A.W. (2021). Recycling and valorization of glass fibre thermoset composite waste by cold incorporation into a sustainable inorganic polymer matrix. Compos. Part B Eng..

[13] Palmer J., Ghita O.R., Savage L., Evans K.E. (2009). Successful closed-loop recycling of thermoset composites. Compos. Part A Appl. Sci. Manuf..

[14] Lewandowski K., Skórczewska K., Piszczek K., Urbaniak W. (2019). Recycled Glass Fibres from Wind Turbines as a Filler for Poly(Vinyl Chloride). Adv. Polym. Technol..

[15] Scaffaro R., Di Bartolo A., Dintcheva N.T. (2021). Matrix and Filler Recycling of Carbon and Glass Fiber-Reinforced Polymer Composites: A Review. Polymers.

[16] Evens T., Bex G.-J., Yigit M., De Keyzer J., Desplentere F., Van Bael A. (2019). The Influence of Mechanical Recycling on Properties in Injection Molding of Fiber-Reinforced Polypropylene. Int. Polym. Process..

[17] Kratz J., Low Y., Fox B. (2017). Resource-friendly carbon fiber composites: Combining production waste with virgin feedstock. Adv. Manuf. Polym. Compos. Sci..

[18] Li Y.-F., Hsu Y.-W., Syu J.-Y., Chen B.-Y., Song B. (2023). Study on the Utilization of Waste Thermoset Glass Fiber-Reinforced Polymer in Normal Strength Concrete and Controlled Low Strength Material. Materials.

[19] Correia J.R., Almeida N.M., Figueira J.R. (2011). Recycling of FRP composites: Reusing fine GFRP waste in concrete mixtures. J. Clean. Prod..

[20] Ribeiro M., Meira-Castro A., Silva F., Santos J., Meixedo J., Fiúza A., Dinis M., Alvim M. (2015). Re-use assessment of thermoset composite wastes as aggregate and filler replacement for concrete-polymer composite materials: A case study regarding GFRP pultrusion wastes. Resour. Conserv. Recycl..

[21] Castro A.M., Ribeiro M., Santos J., Meixedo J., Silva F., Fiúza A., Dinis M., Alvim M. (2013). Sustainable waste recycling solution for the glass fibre reinforced polymer composite materials industry. Constr. Build. Mater..

[22] Conroy A., Halliwell S., Reynolds T. (2006). Composite recycling in the construction industry. Compos. Part A Appl. Sci. Manuf..

[23] Yazdanbakhsh A., Bank L.C. (2014). A Critical Review of Research on Reuse of Mechanically Recycled FRP Production and End-of-Life Waste for Construction. Polymers.

[24] Butenegro J.A., Bahrami M., Swolfs Y., Ivens J., Martínez M.A., Abenojar J. (2022). Novel Thermoplastic Composites Strengthened with Carbon Fiber-Reinforced Epoxy Composite Waste Rods: Development and Characterization. Polymers.

[25] (2012). Plastics—Determination of Tensile Properties—Part 2: Test Conditions for Moulding and Extrusion Plastics.

[26] Hejna A., Barczewski M., Kosmela P., Aniśko J., Mysiukiewicz O., Marć M. (2021). Mandarin peel as an auspicious functional filler for polymer composites. Maced. J. Chem. Chem. Eng..

[27] (2014). Standard Test Method for Tensile Properties of Plastics.

[28] Monticeli F.M., Ornaghi H.L., Voorwald H.J.C., Cioffi M.O.H. (2019). Three-dimensional porosity characterization in carbon/glass fiber epoxy hybrid composites. Compos. Part A Appl. Sci. Manuf..

[29] Lekube B.M., Hermann W., Burgstaller C. (2020). Partially compacted polypropylene glass fiber non-woven composite: Influence of processing, porosity and fiber length on mechanical properties and modeling. Compos. Part A Appl. Sci. Manuf..

[30] Häußler M., Eck M., Rothauer D., Mecking S. (2021). Closed-loop recycling of polyethylene-like materials. Nature.

[31] Pickering S.J. (2006). Recycling technologies for thermoset composite materials—Current status. Compos. Part A Appl. Sci. Manuf..

[32] Barczewski M., Matykiewicz D., Andrzejewski J., Skórczewska K. (2016). Application of waste bulk moulded composite (BMC) as a filler for isotactic polypropylene composites. J. Adv. Res..

[33] Ferreira J.M., Errajhi O.A.Z., Richardson M.O.W. (2006). Thermogravimetric analysis of aluminised E-glass fibre reinforced unsaturated polyester composites. Polym. Test..

[34] López F.A., Martín M.I., Alguacil F.J., Rincón J.M., Centeno T.A., Romero M. (2012). Thermolysis of fibreglass polyester composite and reutilisation of the glass fibre residue to obtain a glass–ceramic material. J. Anal. Appl. Pyrolysis.

[35] Bai Z., Song L., Hu Y., Gong X., Yuen R.K.K. (2014). Investigation on flame retardancy, combustion and pyrolysis behavior of flame retarded unsaturated polyester resin with a star-shaped phosphorus-containing compound. J. Anal. Appl. Pyrolysis.

[36] Heeley E.L., Hughes D.J., Taylor P.G., Bassindale A.R. (2015). Crystallization and morphology development in polyethylene–octakis(n-octadecyldimethylsiloxy)octasilsesquioxane nanocomposite blends. RSC Adv..

[37] Mohammadi M., Yousefi A., Ehsani M. (2012). Study of the thermal and mechanical properties of blown films of high- and low-density polyethylene blends. J. Appl. Polym. Sci..

[38] Pulipati D.P., Jack D.A. (2020). Characterization and Model Validation for Large Format Chopped Fiber, Foamed, Composite Structures Made from Recycled Olefin-Based Polymers. Polymers.

[39] Murat B.I.S., Kamalruzaman M.S., Nor Azman M.H., Misroh M.F. (2020). Assessment of Mechanical Properties of Recycled HDPE and LDPE Plastic Wastes. IOP Conf. Ser. Mater. Sci. Eng..

[40] Amjadi M., Fatemi A. (2020). Tensile Behavior of High-Density Polyethylene Including the Effects of Processing Technique, Thickness, Temperature, and Strain Rate. Polymers.

[41] Blaise A., André S., Delobelle P., Meshaka Y., Cunat C. (2012). Identification of the True Elastic Modulus of High-Density Polyethylene. arXiv.

[42] Farahana R.N., Supri A.G., Teh P.L. (2015). Effect of PVC-MA Coupling Agent on Tensile, Water Absorption and Morphological Properties of Recycled High-Density Polyethylene/Ethylene Vinyl Acetate/Eggshell Powder Composites. J. Adv. Res. Mater. Sci..

[43] Malyutta D.A.I., Matteson K.L., Ryan C., Berry M.P., Bajwa D. (2023). An investigation into the tensile properties of recycled high-density polyethylene (rHDPE) blended with talc filler. Results Mater..

[44] AlMaadeed M.A., Ouederni M., Khanam P.N. (2013). Effect of Chain Structure on the Properties of Glass Fibre/Polyethylene Composites. Mater. Des..

[45] Koffi A., Koffi D., Toubal L. (2021). Mechanical Properties and Drop-Weight Impact Performance of Injection-Molded HDPE/Birch Fiber Composites. Polym. Test..

[46] Bajracharya R.M., Manalo A.C., Karunasena W., Lau K. (2016). Experimental and Theoretical Studies on the Properties of Injection Moulded Glass Fibre Reinforced Mixed Plastics Composites. Compos. Part A Appl. Sci. Manuf..

[47] Bajracharya R.M., Manalo A.C., Karunasena W., Lau K. (2017). Durability characteristics and property prediction of glass fibre reinforced mixed plastics composites. Compos. Part B Eng..

[48] Singleton A.C.N., Baillie C.A., Beaumont P.W.R., Peijs T. (2003). On the Mechanical Properties, Deformation and Fracture of a Natural Fibre/Recycled Polymer Composite. Compos. Part B Eng..

[49] Arunit A., Kers J., Goljandin D., Saarna M., Tall K., Majak J., Herranen H. (2011). Particulate Filled Composite Plastic Materials from Recycled Glass Fibre Reinforced Plastics. Mater. Sci..

[50] Ayadi A., Kraeim D., Bradai C., Pimbert S. (2011). Recycling effect on mechanical behavior of HDPE/glass fibers at low concentrations. J. Thermoplast. Compos. Mater..

[51] Panowicz R., Miedzińska D. (2012). Numerical and experimental research on polyisocyanurate foam. Comput. Mater. Sci..

[52] Qu W., Xue Y., Gao Y., Rover M., Bai X. (2016). Repolymerization of pyrolytic lignin for producing carbon fiber with improved properties. Biomass Bioenergy.

[53] Chen X.-H., Wu G., Chen S.-C., Wang Y.-Z. (2023). Facile, high-efficiency, and low-cost depolymerization of PA6 to ε-caprolactam enables closed-loop chemical recycling. Polymer.

[54] Bajracharya R.M., Manalo A.C., Karunasena W., Lau K. (2014). An Overview of Mechanical Properties and Durability of Glass-Fibre Reinforced Recycled Mixed Plastic Waste Composites. Mater. Des..

[55] Mouzakis D.E., Zoga H., Galiotis C. (2008). Accelerated environmental ageing study of polyester/glass fiber reinforced compo-sites (GFRPCs). Compos. Part B Eng..

